# Sources and selection of snow-specific microbial communities in a Greenlandic sea ice snow cover

**DOI:** 10.1038/s41598-019-38744-y

**Published:** 2019-02-19

**Authors:** Lorrie Maccario, Shelly D. Carpenter, Jody W. Deming, Timothy M. Vogel, Catherine Larose

**Affiliations:** 1 Environmental Microbial Genomics, Laboratoire Ampère, CNRS, École Centrale de Lyon, Écully, France; 2 Microbiology Section, Department of Biology, University of Copenhagen, Copenhagen, Denmark; 3 School of Oceanography, University of Washington, Seattle, USA

## Abstract

Sea ice and its snow cover are critical for global processes including climate regulation and biogeochemical cycles. Despite an increase in studies focused on snow microorganisms, the ecology of snow inhabitants remains unclear. In this study, we investigated sources and selection of a snowpack-specific microbial community by comparing metagenomes from samples collected in a Greenlandic fjord within a vertical profile including atmosphere, snowpack with four distinct layers of snow, sea ice brine and seawater. Microbial communities in all snow layers derived from mixed sources, both marine and terrestrial, and were more similar to atmospheric communities than to sea ice or seawater communities. The surface snow metagenomes were characterized by the occurrence of genes involved in photochemical stress resistance, primary production and metabolism of diverse carbon sources. The basal saline snow layer that was in direct contact with the sea ice surface harbored a higher abundance of cells than the overlying snow layers, with a predominance of *Alteromonadales* and a higher relative abundance of marine representatives. However, the overall taxonomic structure of the saline layer was more similar to that of other snow layers and the atmosphere than to underlying sea ice and seawater. The expulsion of relatively nutrient-rich sea ice brine into basal snow might have stimulated the growth of copiotrophic psychro- and halotolerant snow members. Our study indicates that the size, composition and function of snowpack microbial communities over sea ice were influenced primarily by atmospheric deposition and inflow of sea ice brine and that they form a snow-specific assemblage reflecting the particular environmental conditions of the snowpack habitat.

## Introduction

Sea ice, which forms, grows, melts at the ocean surface and is often overlain by snow can cover up to 22 million km^2^, globally. Snow and ice are not inert barriers, but active interfaces that mediate a range of physico-chemical and microbial reactions involved in global biogeochemical cycles^1–4^. Climate change, amplified in polar regions^5^, influences the extent and duration of the sea ice cover^6^, which in turn strongly affects climate regulation as well as marine ecosystems and global biogeochemical processes^7^.

Snowpacks are formed from accumulated layers of snowflakes that grow in the atmosphere around particles, including microorganisms, that act as ice nucleators leading to snow precipitation^8^. Microbial abundance in snowpack generally ranges from 10^3^ to 10^4^ cells per ml of melted snow^9^. The atmosphere was proposed to be the main seeding source for microorganisms in snow based on several different observations. For example, the snowpacks on ice floes at three sites in the vicinity of the North Pole were shown to harbor similar microbial communities that largely differed from those in the underlying ice and seawater^10^. In a High Canadian Arctic study, the overlap in microbial community composition of air and snow samples with microbial mats in the region suggested the seeding of snow from adjacent cyanobacterial mats via local atmospheric transportation^11^. Dry deposition of long-range-transported atmospheric dust has also been described as a colonization process for non-polar glaciers and remote pristine Antarctica stations^12,13^. Another potential source for microorganisms in snowpacks over seawater is sea ice brine. Elevated microbial abundance has been observed in brine-wetted snow over arctic winter first-year sea ice, and the vast majority of the cells (up to 85%) were intact and functional, based on Live/Dead staining^14^. Upward brine expulsion from the ice matrix was considered to provide biomass to snow, despite potential retention of some microorganisms within the sea ice due to surface adhesion and ice-pore clogging^15–17^.

Within the snowpack, microbes are subjected to a range of physico-chemical conditions related to the specific structure and composition of snow. Snow is highly reactive to UV radiation and defined as a photochemical bioreactor^18,19^. Because radiation and the concomitant hyper-oxidative environment are both deleterious to a wide range of biological molecules, these conditions should influence the composition and physiology of microorganisms inhabiting the snow. While numerous mechanisms for resistance to photo-oxidative stress have been characterized, such as detoxification via antioxidant production or enzymatic action^20,21^ and DNA and protein repair^22,23^, none of these traits has been described in snow microorganisms, with the exception of snow algae^24–29^. The snowpack is also considered to be a low nutrient environment^11,30^. When snow forms, it scavenges organic contaminants and persistent pollutants from anthropogenic sources^31,32^ that might act as carbon sources for snow microbial communities. Previous measurements of dissolved organic carbon (DOC) in non-saline snow over sea ice in the high Arctic have indicated that DOC concentrations are much lower in bulk melted snow (10X less) than in underlying sea ice brine (37.5 mg C L^−1^)^33^, where nutrients and microbial cells are concentrated within the ice matrix^15^. Capillary transport from sea ice brine into snow can bring organic material such as extracellular polymeric substances (EPS)^14^ along with high concentrations of salts^34^. The response to salt stress in a wide range of marine microorganisms involves the transport and metabolism of compatible solutes that are mainly derived from amino acid metabolism and act as osmolytes^35,36^. However, the occurrence of these mechanisms has not been evaluated in snow microbes.

Snowpack microbes have received growing attention in the last decade with studies showing that snow harbors numerous microorganisms that vary in terms of cell abundance, community composition and diversity depending on season, location, depth and other chemical parameters^11,12,37–45^. These findings have contributed to snow microbial communities being considered as part of the cryo-biosphere^9,46,47^, but snowpack microbial ecology remains unclear. Previous suggestions have included that post-depositional processes might lead to the establishment of microbial communities specific to the snow ecosystem^48–50^, further challenging the hypothesis that microbes are simply trapped in a vegetative state in the snowpack before their release to other environments during snowmelt^51^. Our objective was to investigate how selection towards a snow-specific community occurs within snowpacks after deposition. To our knowledge, this study is the first to examine a complete vertical profile of microbial communities structure and potential functions, using high throughput shotgun sequencing, in an ice-covered Greenlandic fjord that includes atmosphere, snowpack, brine within sea ice and seawater. Metagenomes comparison was conducted to test the following hypotheses. First, the snow community would differ from the closely interacting and potential microbial sources: the atmosphere and underlying sea ice. Second, the differences in microbial community structure would relate to the selection of a snow-specific community harboring genetic traits linked to the conditions characteristic of the snowpack on sea ice: the potential to respond to photochemical and osmotic stress in surface and basal snow layer, respectively, as well as different trophic strategies depending on potential nutrient sources.

## Methods

### Site description and sampling procedures

**Figure 1:**
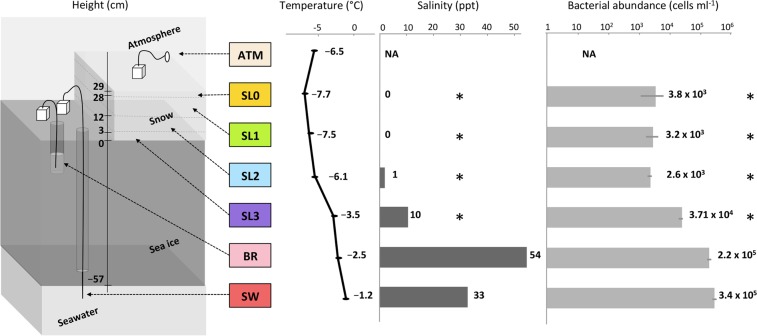
Sampling scheme and sample characteristics. Samples were taken through a vertical profile, from atmosphere (ATM) to four layers of snow (SL0, SL1, SL2, and SL3), including the saline snow layer (SL3), to sea ice brine (BR) taken from a brine-filled sackhole and seawater (SW) below the ice. Color codes for sample type apply to Figs 2, 3, 4, 6 and 7 (and S1). Asterisks indicate measurements made on bulk (melted) snow samples. NA indicates not available.

Samples were collected on 18 March 2014 from a sea ice-covered fjord, Kobbefjord in the vicinity of Nuuk, Greenland (64°07′N, 51°21′W) with no close source of human activity (around 15 km). Due to logistical issues, air sampling was performed on the adjacent coast using a 0.1 μm filter mounted on a Swinnex connected to a remote downstream vacuum pump for the duration of the sampling on the fjord. A 10 m^2^ pristine snowfield with a homogeneous snow cover of 30 cm was selected for triplicate snow pit sampling. A vertical profile was sampled (Fig. 1), with duplicates for the atmosphere (ATM) and triplicate for the snow layers (SL), sea ice brine (BR), and seawater (SW). Four layers (SL0, SL1, SL2, SL3) of the overlying snowpack were identified by visual structure and sampled in 3-L sterile bags: a thin hard top layer in direct contact with the atmosphere (SL0), a basal saline snow layer wetted by an ascending brine flow (SL3), and two intermediate layers (SL1, SL2). Snow-cleared sea ice was drilled with a 9-cm diameter ice corer (Mark II Coring System, Kovac Enterprise) to form a 25-cm deep sackhole and left to fill with brine for one hour and recovered using a 1-L syringe. An adjacent hole was cored to insert tubing, and the underlying seawater was pumped manually. All sampling and storage materials were rinsed with ethanol and a subset of the sampled media (snow, sea ice brine or seawater respectively) prior to sample collection. The temperature was measured for each horizon using an electronic thermometer. Salinity was measured using a portable refractometer. Samples were transported directly to the laboratory and processed at the Greenland Center for Climate Research in Nuuk. Snow samples were melted at room temperature under constant agitation to avoid warming and filtered immediately after melting. All samples were filtered using 0.1 μm filters (Merck Millipore). Filters were stored at −20 °C in an RNA-stable storage reagent (RNAlater, Invitrogen) and shipped to the laboratory in France for further processing. Small portions of liquid samples were fixed with formaldehyde, stored in the cold and dark, and shipped to the University of Washington for bacterial enumeration by epifluorescence microscopy based on the method of Firth *et al*.^52^.

### Molecular biology procedures

Filters and cells possibly detached from the filters were subjected to three successive rinsing/centrifugation steps using a phosphate buffer solution (PBS) to remove the RNAlater reagent. Total nucleic acids were extracted using the Power Water RNA isolation kit (Machery-Nagel), following manufacturer instructions, except that the DNAse treatment step was omitted (DNA and RNA were co-extracted but only the DNA fraction was further analyzed in the present study). Libraries of total genomic DNA were prepared using Nextera XT preparation kit (Illumina) following the manufacturer’s instructions, except that we increased the number of PCR amplifications to 13 cycles as indicated for low DNA input (0.1 ng/µl in our case)^53^. Libraries were purified using AMPure XP beads (New England Biolabs) and their concentration was normalized following Illumina Nextera XT protocol. Bioanalyzer and High Sensitivity DNA Kit (Agilent) were used to check the fragment size distribution of each library. Libraries were sequenced on an Illumina Miseq sequencer based at the Environmental Microbial Genomics group in Laboratoire Ampère (Ecole Centrale de Lyon) with 2 × 250 bp chemistry (MiSeq Reagent Kit v2).

### Bioinformatic analyses

Raw sequencing data were filtered using the base quality upon read length with the fastX toolkit^54^. Due to high variability in sequenced fragment size, forward and reverse reads were not paired and used as technical duplicates (marked with 0.1 or 0.2 at the end of sample name) to verify the congruity of annotation between the forward and reverse reads pools. Metagenome coverage was checked using NonPareil3^55^ based on k-mer redundancy in the datasets and varied from 12% for seawater and up to 35% in surface snow. Quality-checked reads were aligned against the non-redundant (nr) protein reference database using Diamond BlastX algorithm^56^ and alignment outputs were filtered to an e-value threshold of 10e^−5^. Taxonomic composition and functional annotations were analyzed with MEGAN 5^57^. Specifically, taxonomic assignment of each metagenomic read was based on last common ancestor (LCA) of all hits matching NCBI genome database as referenced in MEGAN GI accession number data release from March 2015. Reads with identified protein function (representing on average 35% of all QC-passed reads) were assigned to hierarchical subsystems based on SEED^58^, KEGG^59^, and COG^60^ databases. For specific functions, such as melanin, scytonemin, mycosporine-like amino acids (MAAs) and mannitol biosynthesis, metagenomes were aligned against specific databases created from the NCBI protein database and retrieved using GERMLAB scripts. Microbial attributes were obtained from organisms with described features in NCBI database and referenced in MEGAN5. Trophic life styles were investigated using the ratio of COG functions characteristic of oligotrophy or copiotrophy determined by marine genomes and metagenomes in 2009 by Lauro *et al*.^61^. Statistical analyses were performed using STAMP^62^ and R-packages: ade4tkGui^63^, pvclust^64^, and vegan^65^.

## Results

### Sample characteristics

Salinity corresponded to freshwater (0.5 ppt = g/L) in the upper two layers (SL0, SL1) for bulk (melted) snow and increased with proximity to the sea ice from 1 ppt in the middle snow layer (SL2) to 10 ppt in the basal saline snow layer (SL3), and peaked in the sea ice brine at 54 ppt; seawater salinity was 34 ppt (Fig. 1). No bacterial counts were determined for atmospheric samples due to logistical constraints. The surface snow layers (SL0, SL1 and SL2) contained approximately 3 × 10^3^ cells ml^−1^ of melted snow. Cell abundance was approximately ten times higher in the basal saline snow layer (SL3) (3.7 × 10^4^ cells ml^−1^) than the surface layers and lower than that observed in the sea ice brine and seawater (2.2 and 3.4 × 10^5^ cells ml^−1^ respectively; Fig. 1).

### Taxonomic composition of the microbial communities

**Figure 2:**
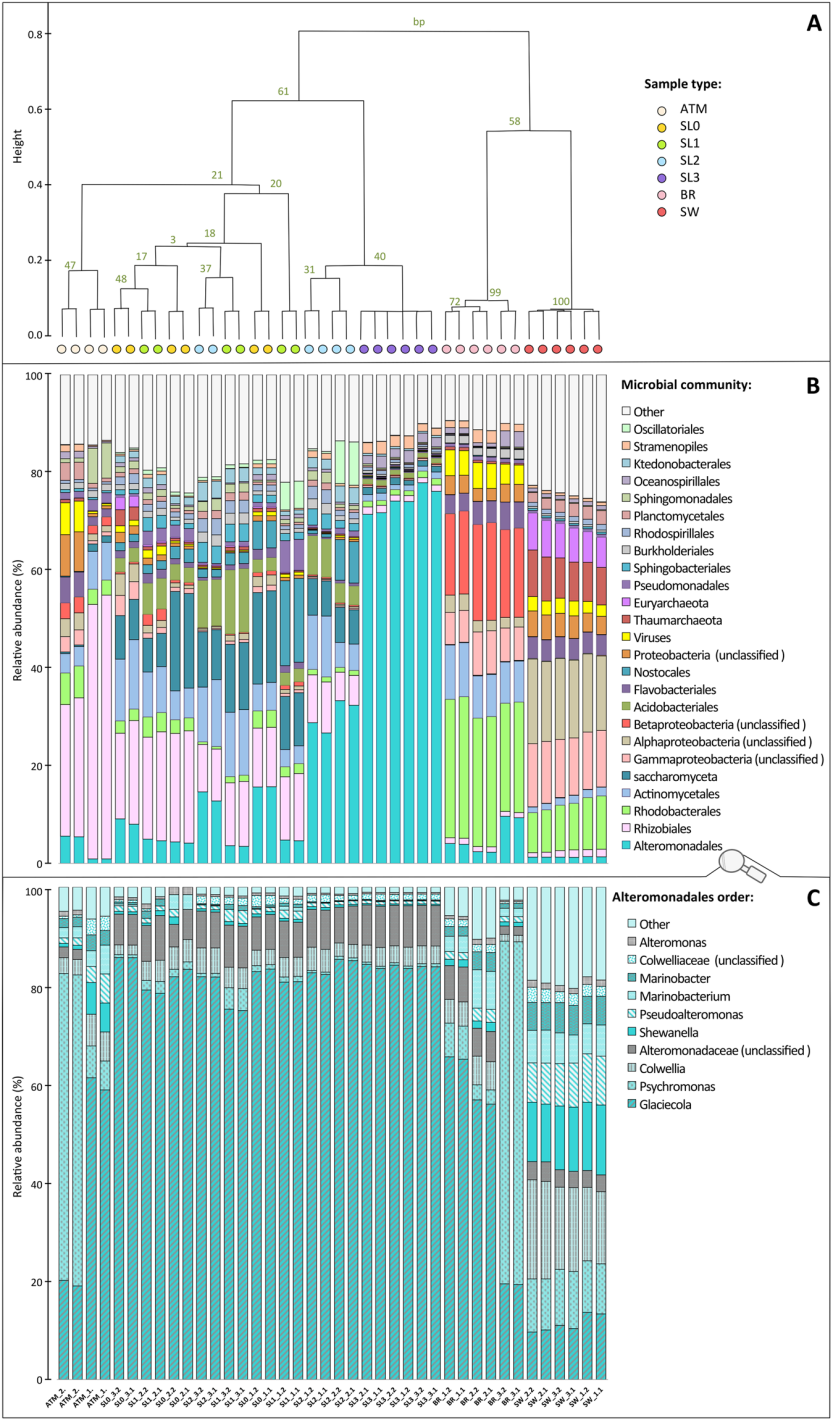
Taxonomic composition. (**A**) Hierarchical cluster using average clustering method and correlation as distance measure and 1000 bootstrap resampling based on relative abundance of all taxa detected. Bootstrap probability (BP) values in percentage are indicated in green. Distance between clusters is indicated by height bar. Graph B and C are organized following the distribution of samples in cluster (colored circles) (**B**) Barplot of relative abundance of 25 most abundant taxa (class or order level, organized by mean decreasing order of abundance from the bottom to the top). (**C**) Barplot of relative abundance of most abundant genus within the *Alteromonadales* order (organized by mean decreasing order of abundance from the bottom to the top).

Clustering of samples based on the relative abundance of taxa retrieved and annotated from the sequences (Fig. 2A) shows two major groups of samples: snow and atmosphere versus seawater and brine, although the snow and atmosphere samples had a greater variation between replicates than the brine and seawater samples. In all samples, the large majority of reads were assigned to prokaryotes (around 90%; Fig. S1), but the twenty-five most detected taxa, which represent between 75% and 90% of taxonomically assigned sequences, included both pro- and eukaryotic representatives (Fig. 2B).

**Figure 3:**
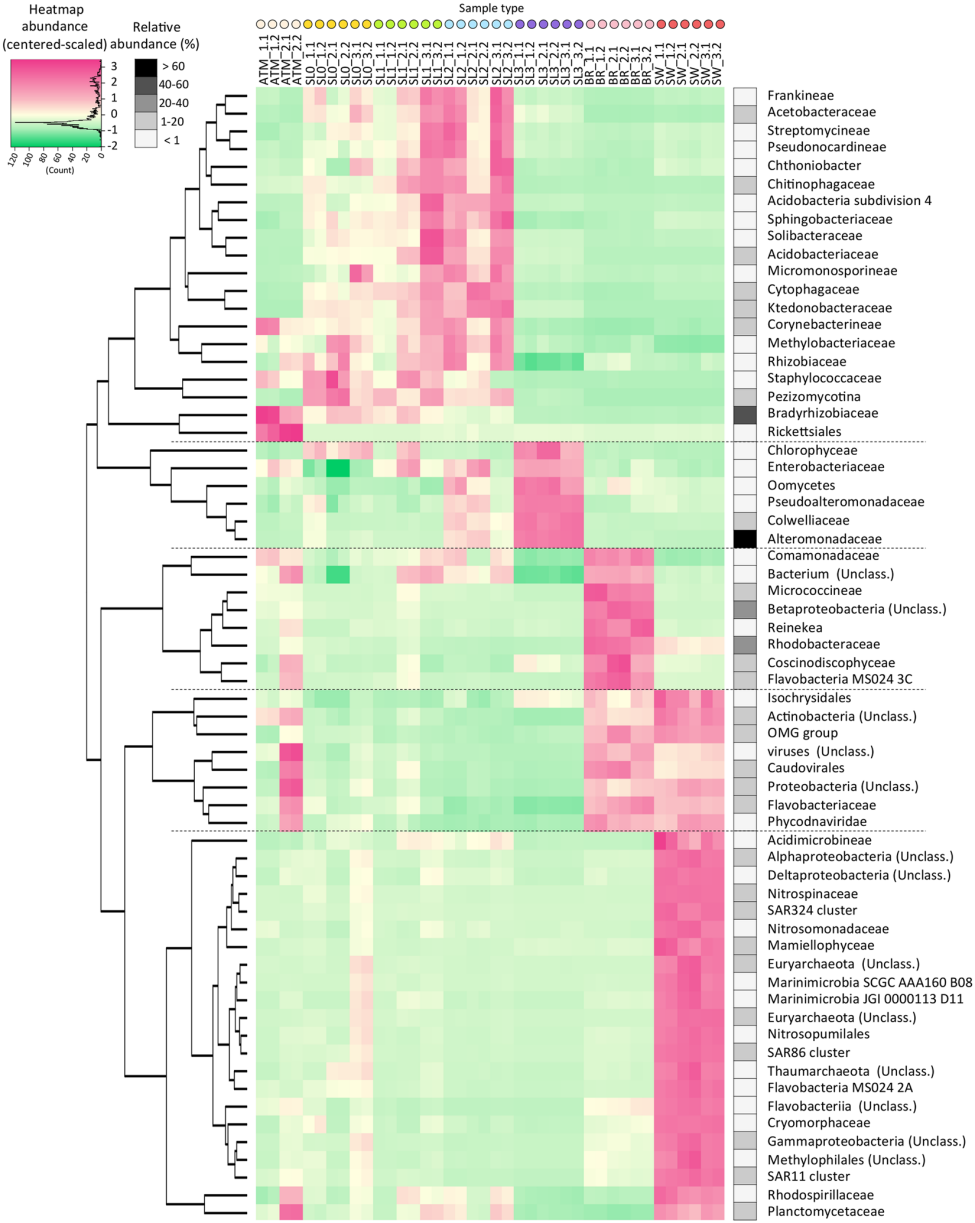
Co-occurrence of taxa retrieved. Heatmap of center-scaled relative abundance of most variable taxa, colored as higher (pink) or lower (green) than the mean observed for all samples (yellow) for each taxon. Most variable taxa were selected as maximum relative abundance observed >0.3% and p value < 10^−5^ using multi group statistical test ANOVA between sample types; surface snow (SL0, SL1, SL2), deep saline snow (SL3), sea ice brine (BR) and seawater (SW) were selected. Hierarchical clustering (using average clustering method and correlation as distance measure and 1000 bootstrap resampling based on correlation of relative abundance of these taxa) was used to investigate co-occurrence of variable taxa. Maximum relative abundance observed in samples for each taxon is indicated in shade of greys.

Atmospheric samples were dominated by *Rhizobiales* (26 to 54% of annotated sequences) and were mostly *Bradyrhizobium*. This taxon was also dominant in the surface snow layers (17% in SL0 and SL1). The surface snow samples (SL0, SL1 and SL2) harbored a high relative abundance of *Saccharomycota*, *Actinomycetales, Alteromonadales, Acidobacteriales*. Cyanobacteria from the order of *Nostocales* were also observed with high relative abundance in some samples (up to 17% in SL1). Within the dominant groups, some families, such as *Ktedonobacteraceae*, *Cytophagaceae* and *Pezizomycotina*, co-occurred in surface snow samples (SL0, SL1 and SL2) with significantly higher relative abundances than in the atmosphere, saline snow, sea ice brine and seawater samples (Fig. 3).

The basal saline snow (SL3) was dominated by *Alteromonadales* (71 to 78% of annotated sequences). The structure of this order was very similar among all snow samples and dominated by *Glaciecola* (about 80%), whereas the atmosphere, sea ice brine and seawater samples harbored numerous other *Alteromonadales* representatives including *Psychromonas*, *Colwellia*, *Shewanella* and *Pseudoalteromonas* (Fig. 2C). SL3 also had higher relative abundances of some *Proteobacteria* (*Oceanospirillaceae*), Protists (*Saprolegniaceae* and *Dictyosteliida*) and algae (*Ectocarpaceae*, *Monodopsidaceae* and *Volvocaceae*) compared to all other samples (Fig. 3). Taxa that were predominant in brine or seawater, such as *Rhodobacter* (about 26%), unclassified *Proteobacteria* (12–18%, *Beta-*, *Gamma*- and *Alpha-*, with mostly SAR11 group), *Thaumarchaeota* (8%) and *Euryarcheota* (7%) were rarely observed in any of the snow layers (Fig. 2B). High abundance of sequences were associated with viruses (mostly *Caudovirales*) in seawater (approx. 2.5%) and in sea ice brine (approx. 5%), but were rarely detected in snow samples.

### General functional profiles of the microbial communities

**Figure 4:**
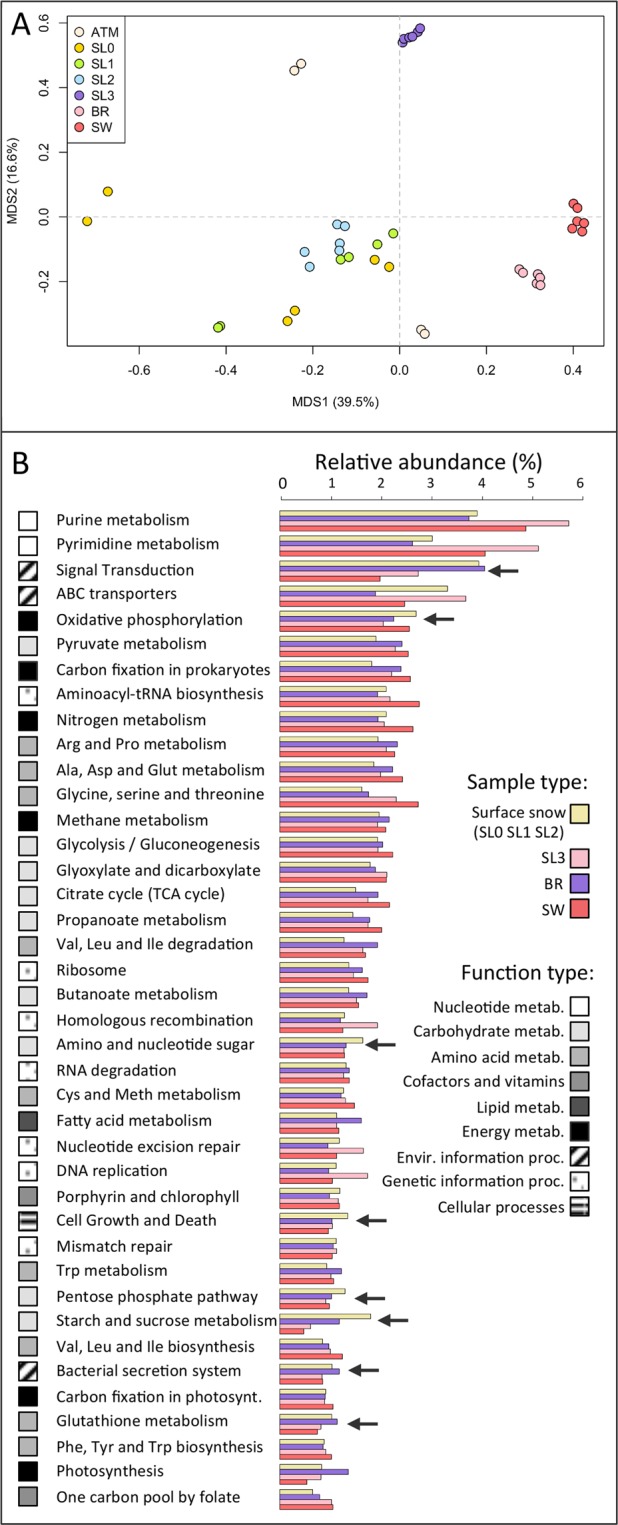
Function distribution. (**A**) Principal coordinates analysis (PCoA) calculated with Bray-Curtis distance on the relative abundance of KEGG functions in each sample. (**B**) Relative abundance of the 40 most abundant KEGG functions organized in decreasing order of abundance (normalized to total abundance of reads annotated in KEGG database, as percentage) in each major group determined by PCoA, Surface snow (SL0, SL1, SL2) saline snow (SL3), sea ice brine (BR) and seawater (SW). Major type of functions (metabolisms of nucleotide, carbohydrate, amino acids, cofactors and vitamins, lipid, as well as energy metabolism, environmental and genetic information processing and cellular processes) are colored in shade of greys. Black arrows indicate functions that were higher in surface and/or saline snow. The variabilities of the represented functions between these sample groups (surface snow, saline snow, brine and seawater) are indicated in Table S2.

**Figure 5:**
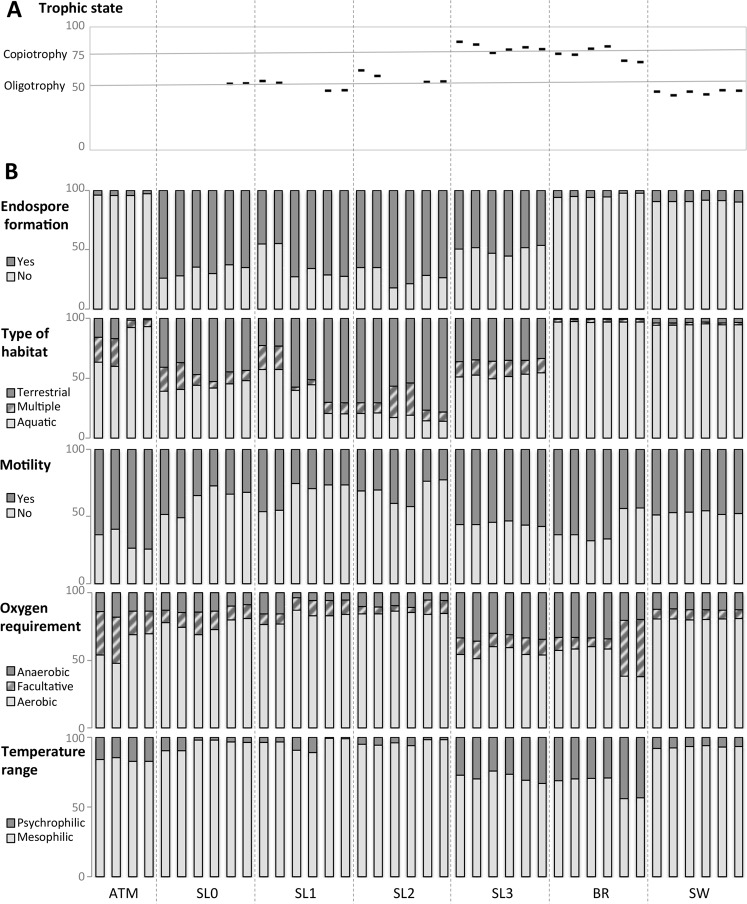
Estimation of microbial attributes. (**A**) Proportion of reads associated with trophic lifestyle (black hyphen), normalized to total abundance of reads annotated for COG categories with variable relative abundance depending on trophic life style (copiotrophic and oligotrophic) as percentage, as determined by comparison of 34 genomes of marine bacteria by Lauro *et al*. (2009). Grey horizontal lines represent the mean proportion of reads associated with copiotrophic life style within the selected COG in oligotrophic organisms (lower line, ratio copio:oligo functions from 50:50) and copiotropic organisms (upper line, ratio copio:oligo functions from 75:25). All samples with less than 100 hits within the selected COG categories were removed from the analysis. The detailed relative abundance of each COG functions investigated is available in Table S2. (**B**) Relative abundance of reads associated with microbial attributes; i.e., reads aligned against a genome obtained from an organism with described features (endospore formation, type of habitat, motility, oxygen requirement, temperature range) obtained from NCBI website and referenced in MEGAN. Read abundance was normalized to total abundance of reads annotated for each microbial attribute, as percentage.

Grouping of samples based on principal coordinates analysis (PCoA) of the relative abundance of functions (185 KEGG categories) produced four major groups related to sample type: seawater, brine, saline snow and a more dispersed group with surface snow samples (SL0, SL1, SL2) and atmosphere together (Fig. 4A). The most dominant observed functions were mainly housekeeping functions and included the metabolism of nucleotides, carbohydrates and amino acids as well as information processing such as signal transduction and ATP-binding cassette (ABC transporters) with significant differences in relative abundance between sample types (Fig. 4B). The comparison of the relative abundance of functions between sample types was also carried out within prokaryotes only (Table S1). Reads associated with signal transduction as well as the metabolism of starch and sucrose, amino sugars, xenobiotics and the biosynthesis of secondary metabolites were enriched in surface and saline snow. Brine and seawater samples exhibited a higher relative abundance of genes involved in amino acid metabolism, in particular glycine and serine, citrate cycle and the metabolism of propanoate and cofactors such as folate. The carbon fixation pathways were more abundant in the saline snow layer, brine and seawater, but all samples harbored primary producers, either *Cyanobacteria*, which were detected in high relative abundance in surface snow, or algae in saline snow, sea ice brine and seawater (Fig. S1). Major differences in principal microbial attributes such as temperature optimum, oxygen requirement, motility, habitat type and the capacity for endospore formation were also observed between sample types (Fig. 5B). Reads associated with mesophilic as well as aerobic life-styles were dominant in all samples, but the proportion of psychrophilic and anaerobic bacteria were higher in the saline snow layer and sea ice brine. These samples also exhibited a higher prevalence of genes related to cell motility. As expected, sea ice brine and seawater samples included reads almost exclusively associated with organisms from aquatic sources, while snow samples, even the basal saline snow layer (SL3), yielded the highest proportions of reads associated with organisms from terrestrial sources (around 40% in SL3 and up to 75% in SL2) and ascribed to multiple habitats (up to 25%).

Based on the ratio of functions characterized as oligotrophic or copiotrophic (derived from marine organism genomes and metagenomes^61^), microbial communities in our surface snow samples (SL0, SL1, SL2) were characterized as oligotrophic and in seawater as extremely oligotrophic, whereas microbial communities from the saline snow layer (SL3) and sea ice brine samples exhibited traits of copiotrophy (Fig. 5A).

### Genomic features of osmotic and photochemical stress responses

**Figure 6:**
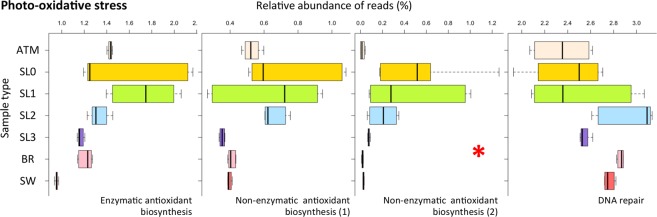
Photochemical stress response. Relative abundance of functions associated with photochemical stress response (UV-radiation and oxidative stress) (normalized to total abundance of annotated reads against SEED database, as percentage). Enzymatic antioxidants include genes coding catalase, superoxide dismutase, rubredoxin, glutaredoxin. Non-enzymatic antioxidant biosynthesis (1) includes genes involved in the biosynthesis of antioxidants and the sunscreens carotenoids, glutathione, mycothiol and tocopherol from SEED subsystems. Non-enzymatic antioxidant biosynthesis (2) refers to scytonemin, melanin, mycosporine like amino-acid (MAA) and mannitol that were identified using a home-made specific database as indicated by the red asterisk. No genes coding for polyol mannitol (mdpa) were detected in any dataset. DNA repair includes genes coding exo- and endonuclease, base excision repair and UVrABC system.

**Figure 7:**
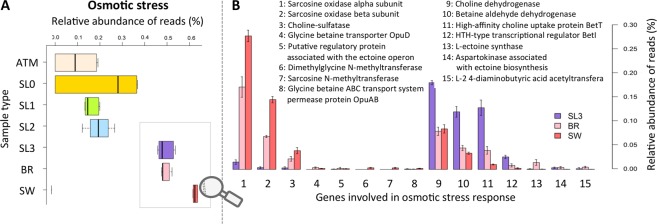
Osmotic stress response. (**A**) Relative abundance of functions associated with osmotic stress responses (annotated according to the SEED subsystems and normalized to total abundance of annotated reads, as percentage). (**B**) Relative abundance of specific osmotic stress responses within saline samples (SL3, BR and SW) (normalized to total abundance of annotated reads, as percentage).

The relative abundance of genes related to photochemical and oxidative stress responses was variable, but generally higher in surface snow layers than in deeper sampling horizons of saline snow, sea ice brine and seawater (Fig. 6). Enzymatic antioxidative mechanisms were significantly more abundant in surface snow layers especially in SL0 and SL1 where they represented up to 2% of total annotated reads. The same trend was observed for non-enzymatic responses. DNA repair mechanisms were present in all sample datasets with similar abundance (between 2 and 3% of the total annotated reads), although at a finer scale, base excision repair was more predominant in surface snow than in other samples (data not shown). The functional group for osmotic stress response mechanisms had a higher relative abundance in saline horizons (SL3, BR and SW at about 0.5%, which is more than twice the amount than in the surface snow; Fig. 7A). Relative abundance of genes associated with choline and bioconversion to glycine betaine was higher in saline snow as compared to brine and seawater. Sarcosine oxidase genes were predominant in brine and seawater and low in snow. Ectoine biosynthesis genes, which were observed less frequently than other compatible solutes (representing less than 0.01% of total annotated reads), had a higher relative abundance in brine and saline snow than in seawater (Fig. 7B).

## Discussion

### From snowflakes in the atmosphere to a snow-specific community selected within snowpacks

Our study confirms that microorganisms in the snow originate from mixed sources with community representatives typically isolated from soil and aquatic habitats, and generalists retrieved from multiple environments. For example, *Segetibacter*, the most abundant genus in our snow samples, belongs to *Sphingobacteriales*, which was previously detected in snow samples^38^ and had been isolated from both soil and the atmosphere^66^. The composition and diversity of atmospheric microbial communities from the same location as in our study, but collected during summer, was correlated with meteorological conditions, highlighting the potential increase of passive bioaerosols from local open soil surfaces with reduced humidity and elevated air temperature during summer^67^. This seasonal finding implies that the contribution from diverse sources and thus the composition of atmospheric microbial communities seeding snow might be highly variable and linked to atmospheric conditions.

Surface snow and atmosphere harbored a more similar community composition as compared to basal snow, but all snow layers and atmosphere communities differed significantly from that of sea ice brine and seawater. Abundant microbes belonging to *Alternomonodales*, *Actinomycetales*, *Saccharomycota*, *Acidobacteriales*, *Nostocales* and *Sphingobacteriales* along with less abundant groups such as *Cytophagales*, *Bacillales*, and *Ktedonobacter* were more prevalent in surface snow as compared to all other sampled horizons. Conversely, *Longameoba*, *Dermacoccaceae*, *Rickettsiales*, *Prochlorococcus and Rhizobiales* decreased in relative abundance from the atmosphere to the snow. Variable ice nucleation abilities in air microbiota that lead to preferential precipitation might explain differences in atmosphere and freshly fallen snow^68^. However, *Rhizobiales* that were predominant in atmosphere samples exhibited a significant drop in snow and decreased with snow depth, despite their potentially high precipitation abilities. *Rhizobiales* were indeed previously detected in similar relative abundances in atmosphere and freshly fallen snow^68^. This taxonomic group was also predominant in young arctic sea ice frost flowers, which are structures that form in part from the condensation of atmospheric supersaturated vapor^69^. As our results thus supported the hypothesis that selection towards snow-specific communities occurs within snowpack, we further investigated the different genetic traits that would enable snow microbes to establish in relation to the unique conditions of the snowpack habitat.

Although UV extinction with snow and sea ice depth was not evaluated in our study, genes involved in resistance to photochemical stress were detected in higher relative abundance in the surface snow layers than in all other horizons and the atmosphere. Microorganisms are also subjected to high radiation in the atmosphere, and cloud microbes that were exposed to UV light in microcosms have been shown to remain metabolically active in the presence of OH radicals^70^. But potentially short residence times of microbes in the atmosphere (3.4 to 7.5 days^71^) might prevent the selection towards microbes with specific genes involved in resistance to photochemical stress. DNA repair mechanisms, widespread among all kingdoms^22^, were detected with similar abundance in all samples, but genes for base excision repair, which is involved during indirect damage to DNA mediated by reactive oxygen species (ROS), were higher in surface snow. The mechanisms identified in our surface snow samples also included a wide range of specific enzymatic responses (*e.g*., catalase, superoxide dismutase) and the production of non-enzymatic sunscreen and antioxidants in photosynthetic organisms (*e.g*., MAA, tocopherol, carotenoids) and non-photosynthetic organisms (*e.g*., melanin, glutathione).

Heterotrophic organisms dominated the microbial communities in all horizons sampled in our study. Surface snow layers exhibited genomic features associated with oligotrophy, even though a greater degree of oligotrophy was observed in the underlying seawater. Surface snow had a higher relative abundance of genes involved in lipid transport and metabolism, cyotochrome P450, and the biosynthesis, transport and catabolism of secondary metabolites. We also observed an increase in gene abundance for carbohydrate metabolic pathways, such as the degradation of starch, sucrose and some xenobiotic compounds, confirming the potential for metabolic versatility of snow microbial communities as highlighted in other studies. For example, microcosms of melted antarctic snow exhibited a higher rate of carbon uptake when amended with a combination of simple and complex carbon sources^72^. The mineralization of radiolabeled 2,4-dichlorophenoxyacetic acid was observed in surface ice cores of the Greenland ice sheet^73^, underlining the involvement of snow microbes in organic carbon contaminant cycling.

Our study supports the hypothesis that primary producers occur within snowpack, composed mostly of *Cyanobacteria* in surface snow and eukaryotic algae in deeper layers (mostly diatoms and *Cryptophytes* for saline snow and sea ice brine and *Prasinophytes* and green algae for seawater). Diverse genes involved in primary production were identified in surface snow samples, although the carbon fixation pathway was less represented in surface snow sequences than in saline snow layer, brine and seawater. However, these processes may be underestimated in surface snow. Along with highly abundant *Nostocales*, numerous sequences were associated with uncharacterized *Cyanobacteria* that were difficult to annotate functionally. Based on our data and those of another study on microbial communities in late spring Greenland sea ice snow^41^, *Cyanobacteria* were the most abundant in the middle layer, which might be due to their development at a depth corresponding to optimum light levels. Distribution of *Cyanobacteria* as a function of snow depth has also been correlated with nitrogen availability in arctic terrestrial spring snow^4^. The distribution of *Cyanobacteria* is largely heterogeneous within the surface layer, where *Nostocales* represented up to 17% of reads in one of our biological replicates, suggesting their development in small patches. Some sequences of non-oxygenic primary producers were also detected (mostly purple sulfur and non-sulfur bacteria with similar abundances in all samples), which is consistent with a previous report suggesting the occurrence of anaerobic niches in the highly porous terrestrial snowpacks^50^.

### Specific niche at the snow–sea ice interface: the saline snow layer

Although the microbial community we observed reflects a snow-specific assemblage that differed from sea ice brine and seawater, basal saline snow shared a critical environmental constraint with the underlying marine horizon: high salinity due to capillary transport from sea ice brine into the basal snow layer. Most of the microbial families detected in higher abundance in the saline snow layer relative to surface snow, such as *Alteromonadaceae*, *Oceanospirillaceae*, *Shewanellaceae*, and *Pseudoalteromonadacea*, include halotolerant marine representatives. In a concurrent study measuring ^14^C-labelled choline uptake, sea ice microbial communities were observed to transport and retain ^14^C-solutes as intracellular osmolytes when salinity was increased^52^. The rise in relative abundance of the genes involved in choline uptake and bioconversion to the osmoprotectant glycine betaine that we observed in the saline snow layer microbial community (compared to surface snow and even to sea ice brine and seawater) is consistent with the use of compatible solutes for osmoprotection. The most abundant genus in the saline snow, *Glaciecola*, lacks the catabolic pathway of sarcosine oxidase, and this pathway and general amino acid metabolism were underrepresented in the basal snow as compared to the brine and seawater. While the genes encoding choline uptake and bioconversion are widespread in halotolerant microorganisms, the capacity to further catabolize glycine betaine via sarcosine oxidase is rarer and may represent a long-term adaptation to the sea ice environment^74^.

The microbial community in the saline basal snow layer exhibited numerous genetic features of copiotrophy, a higher microbial biomass levels (based on cell counts) relative to surface snow layers and an increase in representatives described as psychrophiles, as found in the sea ice brine microbial community. Although we did not measure DOC in our samples, we hypothesize that brine capillarity into the basal snow layer delivers not only salts but also nutrients that stimulate the growth of organisms already present in the snow and lead to their selection. In particular, halotolerant, copiotrophic, psychrophilic or psychrotolerant snow organisms, such as belonging to the group of *Alteromonadales*, might be at a competitive advantage to maintain elevated metabolic rates despite cold temperatures and high salinity. Numerous predominant taxa in sea ice brine samples, such as *Rhodobacterales* and its most represented genus *Octadecabacter*, were far less frequent in saline snow than in brine despite demonstrating many genomic features of adaptation to icy, saline and polar habitats^75,76^. Further studies are needed to decipher if the source of saline snow community is an enrichment of snow microbes, upward seeding from sea-ice brine or a combination of both.

Sequences annotated as viruses were detected in high abundance in seawater and sea ice brine, representing up to 2.5 and 5% of the reads, respectively, while less than 0.1% in saline snow. Genes involved in DNA metabolism (including DNA polymerase, recycling of host nucleotides, and de novo synthesis via ribonucleotide reductase) were the most virally enriched auxiliary metabolic genes (acquired from host genomes) in sea ice brine and seawater datasets, as previously observed in temperate ocean metagenomes^77,78^. A parallel study carried out at our study site using epifluorescence microscopy confirmed elevated densities of viruses and high virus-to-bacteria ratios in both sea ice brine and seawater^52^. Virus-host interactions are thought to be a key feature of sea ice ecosystems, with a major role of viruses in the control of bacterial populations and nutrient turnover^79–81^. Our metagenomic results, however, did not provide evidence of the transport of viruses from sea ice brine into snow or of viral reproduction in the saline snow layer, leaving the importance of viruses in snow microbial ecology completely unknown.

## Conclusion

**Figure 8:**
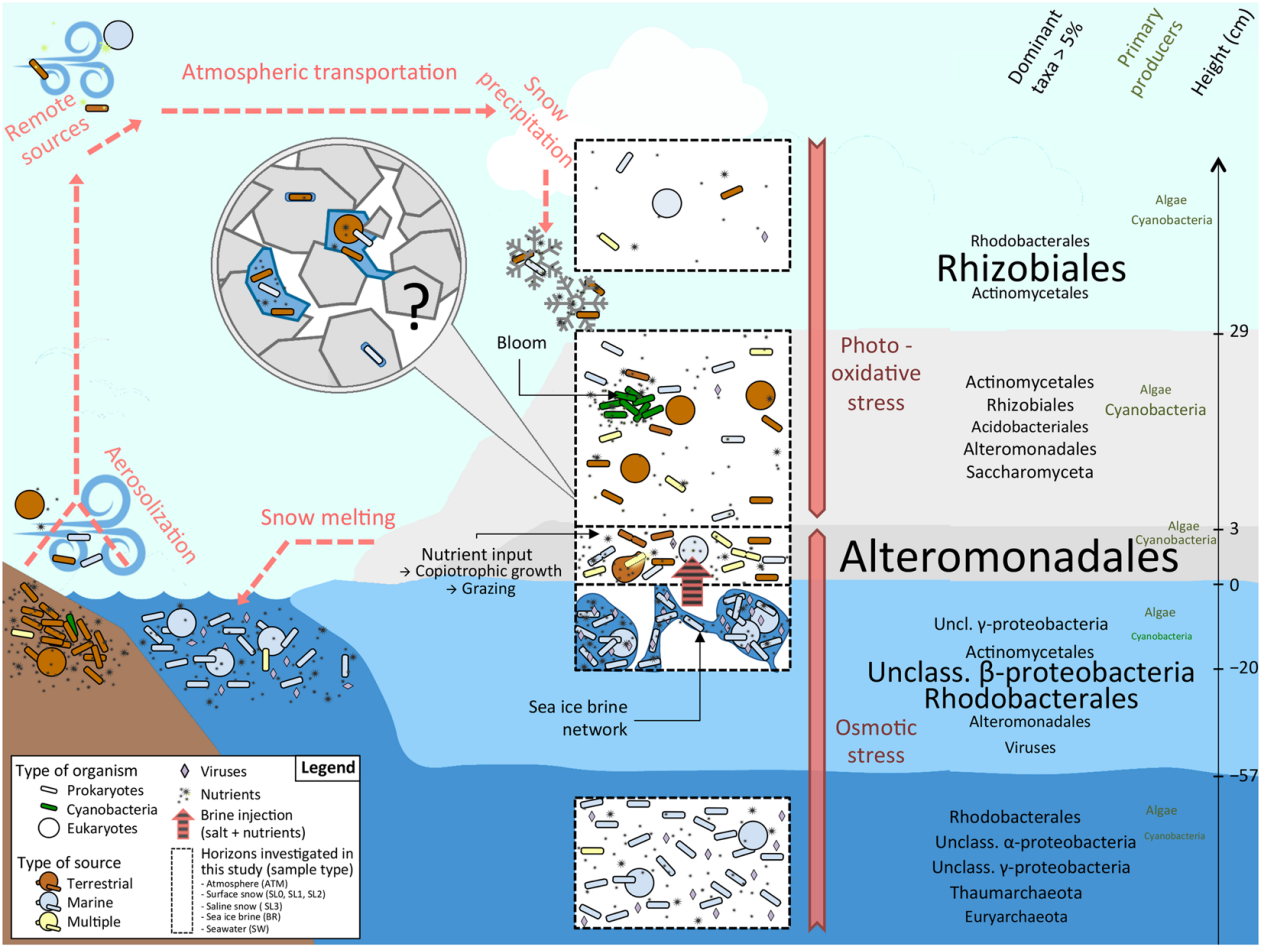
Synthetic diagram of putative microbial ecology of sea ice snow cover. The snow microbe cycle is represented with red dashed arrows; microorganisms along with nutrients are aerosolized from terrestrial and marine environments from local or remote sources, transported atmospherically, precipitated during snowfall and finally released to other environment through melt. Horizons investigated in our study include atmosphere (ATM), surface snow (SL0, SL1, SL2), saline snow (SL3), sea ice brine (BR), and seawater (SW), highlighted in black dashed-line rectangles. Snow harbors a mix of microbes from terrestrial (brown) and marine (blue) origin as well as numerous representatives retrieved in multiple environments (yellow). In surface snow, *Cyanobacteria* (green) might develop in small patches. In basal snow, sea ice brine injection might provide salt and nutrients leading to the growth of copiotrophic, psychro- and halotolerant organisms, especially from the γ-proteobacteria Alteromonadales. The microstructure of the snow ecosystem, including microbe localization and concentration (within the ice crystals or in liquid layer around), the occurrence of anaerobic niches, and microbial interactions influencing individual processes remains unknown (black question mark). Stresses specific to the different horizons (photooxidative stress in surface and osmotic stress in deeper layers) are indicated in orange. Dominant taxa (with relative abundance higher than 5%) and primary producers are specified to the right of the figure; font size is proportional to the relative abundance of each taxon.

The Greenland fjord snow–ice microbial communities that we studied were strongly influenced by their atmospheric seeding sources, with organisms originating from terrestrial and marine environments as well as generalists retrieved ubiquitously. We inferred that post-depositional selection processes occurred in the snow to form a snow-specific microbial community (Fig. 8). Composed of bacteria and eukaryotes (mostly fungi), while archaea and viruses were mostly absent, these communities were characterized by resistance to photochemical and osmotic stress in surface and basal snow layers, respectively. This study improved our understanding of the trophic strategies of snowpack microbial communities. The occurrence of *Cyanobacteria*, likely blooming in patches in surface snow, and of diatoms in saline snow, as well as some non-oxygenic producers, highlighted the potential for primary production. A high versatility in carbon source utilization within the predominant heterotrophic community was also observed. The saline snow layer, potentially fed with nutrients from the upward expulsion of sea ice brine, likely supported the growth of copiotrophic, psychro- and halotolerant snow members and could represent a unique niche with elevated microbial activity. Numerous questions remain as the spatial and time scales of snow microbial life cannot yet be compared to the high chemical and physical dynamics of Arctic snowpacks, limiting our understanding of snow microbial processes. A combination of microbiological data with detailed chemical profiles, measured in sufficient resolution to circumvent the highly variable structure of snowpack, will be necessary to further address the contributions of snow-specific microbial communities to large-scale geochemical cycles and evaluate the impact of their potential loss due to climate change.

## Supplementary information

Supplementary Information

## Data Availability

All metagenomes are available on Mg-Rast server under the Accession Number 4620817.3 to 4620838.3 (www.mg-rast.org).
