# Supplementary material for: Sources and selection of snow-specific microbial communities in a Greenlandic sea ice snow cover
Source: Sci Rep. 2019 Feb 19;9:2290. doi: 10.1038/s41598-019-38744-y (PMC6381142; doi:10.1038/s41598-019-38744-y)
Supplement: Supplementary file 1 — Supplementary Information [file 41598_2019_38744_MOESM1_ESM.pdf]

Scientific Reports

Supplementary information for

Sources and selection of snow-specific microbial communities in a Greenlandic sea ice snow cover

Lorrie Maccario[1,2], Shelly D. Carpenter[3], Jody W. Deming[3], Timothy M. Vogel[1], Catherine Larose[1]

[1] Environmental Microbial Genomics, Laboratoire Ampère, CNRS, École Centrale de Lyon, Écully, France.
[2] Microbiology Section, Department of Biology, University of Copenhagen, Copenhagen, Denmark
[3] School of Oceanography, University of Washington, Seattle, USA

Corresponding Author: Lorrie Maccario - lorrie.maccario@bio.ku.dk

Figure S1: Taxonomic composition. Detailed taxonomic composition at kingdom level (A) and for prokaryotes (B), eukaryotes (C) and primary producers (D) (normalized to total abundance of annotated reads, as percentage).

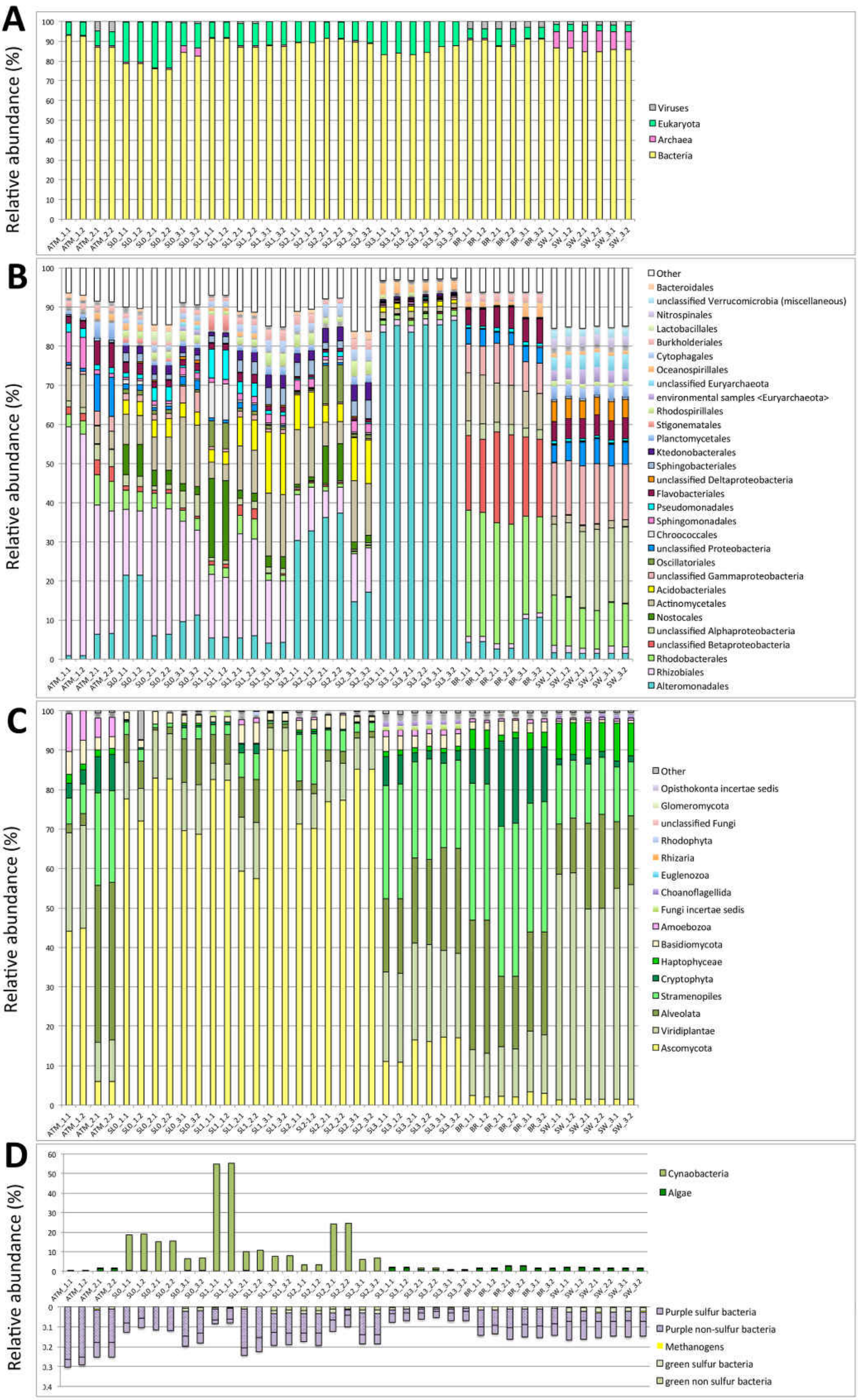
A
B
C
D
Relative abundance (%)
Viruses
Eukaryota
Archaea
Bacteria
Other
Bacteroidales
unclassified Verrucomicrobia (miscellaneous)
Nitrospinales
Lactobacillales
Burkholderiales
Cytophagales
Oceanospirillales
unclassified Euryarchaeota
environmental samples <Euryarchaeota>
Rhodospirillales
Stigonematales
Planctomycetales
Ktedonobacterales
Sphingobacteriales
unclassified Deltaproteobacteria
Flavobacteriales
Pseudomonadales
Sphingomonadales
Chroococcales
unclassified Proteobacteria
Oscillatoriales
unclassified Gammaproteobacteria
Acidobacteriales
Actinomycetales
Nostocales
unclassified Alphaproteobacteria
unclassified Betaproteobacteria
Rhodobacterales
Rhizobiales
Alteromonadales
Opisthokonta incertae sedis
Glomeromycota
unclassified Fungi
Rhodophyta
Rhizaria
Euglenozoa
Choanoflagellida
Fungi incertae sedis
Amoebozoa
Basidiomycota
Haptophyceae
Cryptophyta
Stramenopiles
Alveolata
Viridiplantae
Ascomycota
Cynaobacteria
Algae
Purple sulfur bacteria
Purple non-sulfur bacteria
Methanogens
green sulfur bacteria
green non sulfur bacteria
ATM_1.1
ATM_1.2
ATM_2.1
ATM_2.2
SL0_1.1
SL0_1.2
SL0_2.1
SL0_2.2
SL0_3.1
SL0_3.2
SL1_1.1
SL1_1.2
SL1_2.1
SL1_2.2
SL1_3.1
SL1_3.2
SL2_1.1
SL2_1.2
SL2_2.1
SL2_2.2
SL2_3.1
SL2_3.2
SL3_1.1
SL3_1.2
SL3_2.1
SL3_2.2
SL3_3.1
SL3_3.2
BR_1.1
BR_1.2
BR_2.1
BR_2.2
BR_3.1
BR_3.2
SW_1.1
SW_1.2
SW_2.1
SW_2.2
SW_3.1
SW_3.2

		BACTERIA										
		**p-values**	**ATM: mean**	**ATM: std**	**SL: mean**	**SL: std**	**SL3: mean**	**SL3: std**	**BR: mean**	**BR: std**	**SW: mean**	**SW: std**
Amino Acid Metabolism	Lysine degradation	1.97E-13	0.842	0.071	0.769	0.032	0.988	0.024	0.951	0.028	1.021	0.019
Amino Acid Metabolism	Glycine, serine and threonine metabolism	1.19E-10	1.986	0.070	1.764	0.343	1.961	0.059	2.666	0.067	3.033	0.024
Amino Acid Metabolism	Histidine metabolism	1.20E-08	0.737	0.091	0.715	0.048	0.838	0.004	0.863	0.009	0.925	0.026
Amino Acid Metabolism	Valine, leucine and isoleucine biosynthesis	7.15E-08	0.887	0.076	0.943	0.114	1.066	0.036	1.131	0.029	1.273	0.023
Amino Acid Metabolism	Valine, leucine and isoleucine degradation	2.03E-07	1.948	0.305	1.456	0.183	2.158	0.010	1.856	0.061	1.754	0.047
Amino Acid Metabolism	Arginine and proline metabolism	1.19E-06	1.637	0.429	2.204	0.196	2.610	0.013	2.424	0.034	2.498	0.030
Amino Acid Metabolism	Alanine, aspartate and glutamate metabolism	1.57E-06	1.793	0.326	2.139	0.203	2.504	0.023	2.273	0.028	2.587	0.022
Amino Acid Metabolism	Phenylalanine metabolism	3.14E-05	1.113	0.138	0.859	0.111	0.776	0.015	1.018	0.015	0.867	0.022
Biosynthesis of Other Secondary Metabolites	Biosynthesis of Other Secondary Metabolites	6.52E-07	1.194	0.075	1.330	0.178	1.127	0.032	0.944	0.016	0.904	0.023
Carbohydrate Metabolism	Starch and sucrose metabolism	1.05E-12	1.124	0.058	1.879	0.317	1.325	0.020	0.694	0.076	0.484	0.018
Carbohydrate Metabolism	Propanoate metabolism	1.88E-10	1.965	0.127	1.696	0.103	1.989	0.057	2.003	0.020	2.176	0.024
Carbohydrate Metabolism	Amino sugar and nucleotide sugar metabolism	4.30E-08	1.330	0.227	1.778	0.126	1.483	0.031	1.389	0.020	1.339	0.038
Carbohydrate Metabolism	Citrate cycle (TCA cycle)	7.57E-08	1.879	0.031	1.676	0.217	2.159	0.021	1.974	0.010	2.261	0.032
Carbohydrate Metabolism	Galactose metabolism	1.26E-07	0.408	0.087	0.704	0.127	0.432	0.012	0.437	0.047	0.381	0.013
Carbohydrate Metabolism	Ascorbate and aldarate metabolism	8.77E-06	0.381	0.042	0.344	0.086	0.187	0.012	0.307	0.011	0.430	0.006
Carbohydrate Metabolism	Pyruvate metabolism	1.01E-05	2.202	0.096	2.148	0.335	2.740	0.018	2.626	0.041	2.752	0.030
Carbohydrate Metabolism	Inositol phosphate metabolism	1.67E-05	0.139	0.040	0.281	0.084	0.168	0.014	0.267	0.003	0.352	0.014
Carbohydrate Metabolism	C5-Branched dibasic acid metabolism	2.59E-05	0.403	0.062	0.445	0.097	0.460	0.021	0.549	0.016	0.640	0.016
Carbohydrate Metabolism	Glycolysis / Gluconeogenesis	8.67E-05	2.010	0.077	2.057	0.164	2.243	0.022	2.210	0.071	2.364	0.034
Cell Growth and Death	Cell Growth and Death	2.10E-06	1.532	0.298	1.025	0.151	0.805	0.025	0.932	0.019	0.976	0.024
Cell Motility	Cell Motility	2.84E-15	1.250	0.163	1.173	0.196	2.132	0.088	1.543	0.122	0.392	0.019
Energy Metabolism	Carbon fixation pathways in prokaryotes	9.79E-10	2.358	0.047	2.076	0.201	2.728	0.037	2.549	0.011	2.622	0.017
Lipid Metabolism	Sphingolipid metabolism	8.16E-14	0.258	0.045	0.296	0.041	0.070	0.005	0.082	0.012	0.176	0.010
Lipid Metabolism	Arachidonic acid metabolism	3.06E-10	0.189	0.015	0.192	0.025	0.237	0.005	0.114	0.011	0.146	0.006
Lipid Metabolism	Fatty acid metabolism	6.38E-08	1.679	0.253	1.341	0.127	1.823	0.010	1.296	0.024	1.266	0.032
Lipid Metabolism	Fatty acid biosynthesis	1.27E-06	0.960	0.210	0.768	0.099	0.535	0.011	0.643	0.020	0.929	0.020
Lipid Metabolism	Synthesis and degradation of ketone bodies	2.21E-05	0.403	0.041	0.293	0.065	0.419	0.021	0.356	0.013	0.277	0.008
Lipid Metabolism	Biosynthesis of unsaturated fatty acids	6.28E-05	0.466	0.118	0.421	0.060	0.373	0.011	0.297	0.008	0.286	0.009
Membrane Transport	Membrane Transport	1.81E-07	6.527	0.142	5.462	0.933	3.622	0.072	5.438	0.071	3.893	0.077
Metabolism of Cofactors and Vitamins	One carbon pool by folate	4.77E-07	0.909	0.041	0.688	0.162	0.860	0.026	0.958	0.022	1.116	0.009
Metabolism of Cofactors and Vitamins	Folate biosynthesis	9.13E-07	0.379	0.029	0.497	0.113	0.698	0.019	0.414	0.019	0.367	0.006
Metabolism of Cofactors and Vitamins	Biotin metabolism	3.06E-05	0.354	0.149	0.141	0.060	0.217	0.003	0.105	0.011	0.107	0.007
Metabolism of Other Amino Acids	beta-Alanine metabolism	9.95E-10	0.696	0.016	0.639	0.058	0.775	0.019	0.687	0.012	0.848	0.013
Metabolism of Other Amino Acids	Cyanoamino acid metabolism	5.77E-07	0.444	0.108	0.451	0.079	0.574	0.025	0.267	0.013	0.324	0.009
Metabolism of Other Amino Acids	Taurine and hypotaurine metabolism	6.60E-07	0.315	0.113	0.431	0.032	0.560	0.005	0.378	0.031	0.469	0.009
Nucleotide Metabolism	Pyrimidine metabolism	5.12E-09	3.767	0.609	3.072	0.211	2.674	0.072	3.942	0.087	3.954	0.058
Signal Transduction	Signal Transduction	3.19E-12	4.085	0.351	4.065	0.432	4.085	0.045	2.984	0.088	2.073	0.024
Translation	Translation	5.27E-05	3.524	0.092	3.691	0.546	3.714	0.070	4.044	0.072	4.719	0.043
Transport and Catabolism	Transport and Catabolism	2.97E-05	0.850	0.045	0.744	0.132	0.641	0.007	0.535	0.020	0.573	0.011
Xenobiotics Biodegradation and Metabolism	Xenobiotics Biodegradation and Metabolism	1.88E-05	3.912	0.707	3.326	0.330	2.990	0.029	2.749	0.008	2.612	0.077

		EUKARYA										
		p-values	ATM: mean	ATM: std	SL: mean	SL: std	SL3: mean	SL3: std	BR: mean	BR: std	SW: mean	SW: std
Amino Acid Metabolism	Phenylalanine metabolism	5.63E-10	0.213	0.007	0.601	0.116	0.331	0.115	0.168	0.032	0.276	0.089
Biosynthesis of Other Secondary Metabolites	Biosynthesis of Other Secondary Metabolites	3.45E-05	0.375	0.065	1.231	0.387	0.792	0.152	0.606	0.058	0.647	0.172
Carbohydrate Metabolism	C5-Branched dibasic acid metabolism	1.15E-11	0.753	0.236	0.094	0.058	0.140	0.071	0.449	0.104	0.098	0.059
Carbohydrate Metabolism	Amino sugar and nucleotide sugar metabolism	2.37E-07	0.478	0.038	1.499	0.325	0.827	0.187	0.724	0.154	1.203	0.200
Cell Communication	Cell Communication	1.10E-06	2.122	0.254	1.030	0.347	1.771	0.071	1.650	0.326	1.794	0.264
Energy Metabolism	Carbon fixation in photosynthetic organisms	3.66E-09	0.798	0.029	0.489	0.232	1.164	0.044	1.108	0.162	1.241	0.250
Energy Metabolism	Oxidative phosphorylation	1.19E-05	6.341	0.143	5.831	1.622	4.564	0.129	3.927	0.199	2.451	0.214
Energy Metabolism	Nitrogen metabolism	8.51E-05	1.657	0.211	0.755	0.384	1.461	0.291	1.337	0.243	1.132	0.164
Glycan Biosynthesis and Metabolism	Glycan Biosynthesis and Metabolism	8.41E-07	0.795	0.135	2.685	0.756	1.782	0.098	0.927	0.255	1.630	0.307
Lipid Metabolism	Glycerophospholipid metabolism	7.21E-06	0.959	0.030	1.541	0.545	0.639	0.115	0.378	0.088	0.999	0.255
Membrane Transport	Membrane Transport	1.74E-14	2.927	0.069	0.518	0.290	1.200	0.120	1.884	0.336	1.198	0.097
Metabolism of Cofactors and Vitamins	Porphyrin and chlorophyll metabolism	2.96E-06	1.166	0.177	0.439	0.309	0.732	0.100	0.819	0.162	1.202	0.172
Metabolism of Other Amino Acids	Selenocompound metabolism	2.71E-05	0.801	0.078	0.303	0.212	0.814	0.144	0.421	0.227	0.710	0.205
Metabolism of Terpenoids and Polyketides	Metabolism of Terpenoids and Polyketides	3.44E-07	0.375	0.065	1.009	0.208	1.036	0.083	0.630	0.136	1.225	0.091
Nucleotide Metabolism	Pyrimidine metabolism	1.36E-13	5.582	0.307	2.763	0.493	2.891	0.267	5.205	0.235	3.543	0.388
Nucleotide Metabolism	Purine metabolism	7.39E-08	5.375	0.100	3.763	0.765	3.665	0.285	5.917	0.153	4.395	0.352
Replication and Repair	Replication and Repair	8.53E-08	2.490	0.402	4.832	0.894	3.063	0.142	2.616	0.263	4.167	0.354
Signal Transduction	Signal Transduction	2.97E-05	6.379	0.335	4.658	0.838	6.293	0.321	5.975	0.847	6.030	0.199
Transcription	Transcription	1.35E-07	7.029	0.004	4.436	0.689	4.802	0.525	6.164	0.467	4.443	0.191

Table S1: Variability of the distribution of metabolic functions within sample groups. Comparisons by ANOVA were performed in order to identify functions (based on KEGG database annotation) with significant difference between samples groups: atmosphere (ATM), surface snow (SL0, SL1 and SL2), sea ice brine (BR) and seawater (SW). Mean and standard deviation of relative abundance of each function in each group as well as p-values of the test are indicated. Only functions with relative abundance > 0.1 % and with p-values < 0.0001 are listed.

Genomic features associated with trophic strategies as defined by Lauro *et al.* (2009)			Comparison of relative abundance in our samples				
Markers	Copiotroph	Oligotroph	Surface snow	Deep snow	Brine	Seawater	p-values
COG3386 (gluconolactonase)	Low (0%)	High (0.065%)	0.0349	0.0387	0	0	0.7705
COG1804 (predicted acyl-CoA transferases/carnitine dehydratase)	Low (0%)	High (0.072%)	0.0162	0	0.0188	0.0152	0.042
COG3293 (transposase and inactivated derivatives)	Low (0%)	High (0.073%)	0	0	0	0.0056	0.227
COG2124 (cytochrome P450)	Low (0%)	High (0.078%)	0.4501	0.1962	0.0286	0.1598	0.0164
COG1228 (imidazolonepropionase and related amidohydrolases)	Low (0.064%)	High (0.089%)	0	0.0043	0	0	0.227
COG0483 (archaeal fructose-1,6-bisphosphatase and related enzymes of inositol monophosphatase family)	Low (0.033%)	High (0.115%)	0.021	0.0145	0	0.069	0.004
COG1680 (β -lactamase class C and other penicillin binding proteins)	Low (0.059%)	High (0.115%)	0.0144	0.0197	0	0.0282	0.3453
COG0318 (Acyl-CoA synthetases [AMP-forming/AMP-acid ligases II)	Low (0.149%)	High (0.235%)	0.7389	0.1132	0.1695	0.2257	0.3634
COG1024 (enoyl-CoA hydratase/carnithine racemase)	Low (0.149%)	High (0.308%)	0.1245	0.0187	0	0.0503	0.4735
COG0625 (GST)	Low (0.298%)	High (0.342%)	0.0433	0.0334	0.0515	0.0424	0.5324
COG1960 (acyl-CoA dehydrogenases)	Low (0.186%)	High (0.417%)	0.131	0.1445	0.2902	0.4398	0.0002
COG0596 (predicted hydrolases or acyltransferases: α /β hydrolase superfamily)	Low (0.327%)	High (0.514%)	0.1585	0.0898	0.1687	0.1657	0.1694
COG1028 (dehydrogenases with different specificities: related to short-chain alcohol dehydrogenases)	Low (0.476%)	High (1.135%)	1.0019	0.1708	0.2788	0.7821	0.4607
COG0183 (acetyl-CoA acetyltransferase)	Low (0.114%)	High (1.171%)	0.1323	0.0282	0.1992	0.3869	0.0428
COG category Q (secondary metabolites biosynthesis, transport, and catabolism)	Low (2.173%)	High (3.544%)	2.5371	0.8922	1.0205	2.3367	0.0092
COG category I (Lipid transport and metabolism)	Low (2.958%)	High (4.408%)	3.6829	1.4936	2.0434	3.4013	2.00E-06
COG0814 (amino acid permeases)	High (0.114%)	Low (0%)	0.2164	0.1468	0.1012	0.007	0.597
COG3325 (chitinase)	High (0.06%)	Low (0%)	0.038	0.01	0	0	0.2611
COG0737 (5′-nucleotidase/2′,3′-cyclic phosphodiesterase and related esterases)	High (0.098%)	Low (0.035%)	0.2849	0.019	0	0.0056	0.5606
COG2200 (FOG:EAL domain)	High (0.372%)	Low (0.048%)	0	0.0332	0	0	2.00E-06
COG0243 (anaerobic dehydrogenases, typically selenocysteine-containing)	High (0.179%)	Low (0.0675%)	0.0026	0	0	0	0.6693
COG0110 (acetyltransferase: isoleucine patch superfamily)	High (0.166%)	Low (0.068%)	0.0431	0	0	0	0.5158
COG0583 (transcriptional regulator)	High (1.489%)	Low (0.371%)	0	0	0	0.0158	0.0129
COG category N (cell motility)	High (3.311%)	Low (1.006%)	0.0324	0.1559	0.0518	0.0292	0.002
COG category V (defense mechanisms)	High (1.474%)	Low (1.236%)	0.8221	0.9221	0.7071	0.8415	0.2746
COG category T (signal transduction mechanisms)	High (7.071%)	Low (3.632%)	1.9456	4.7023	2.7709	1.4747	6.00E-08
COG category K (transcription)	High (7.527%)	Low (6.621%)	2.7798	2.2183	4.7613	2.2745	1.00E-10

Table S2: Genomic features associated with trophic strategies. Genes (as indexed in the COG database) associated with a specific trophic lifestyle, oligotrophy or copiotrophy, as determined in the study by Lauro and colleagues (Lauro et al., 2009) from genomic and metagenomic comparisons in various marine environments that were retrieved in our datasets.
